# Recent Trends in the Management of Varicocele

**DOI:** 10.3390/jcm14155445

**Published:** 2025-08-02

**Authors:** Tamás Takács, Anett Szabó, Zsolt Kopa

**Affiliations:** 1 Centre of Andrology, Department of Urology, Semmelweis University, 1082 Budapest, Hungary; a.szabo1995@gmail.com (A.S.); kopaandro@gmail.com (Z.K.); 2 Centre of Assisted Reproduction, Department of Obstetrics and Gynaecology, Semmelweis University, 1082 Budapest, Hungary; 3 Centre for Translational Medicine, Semmelweis University, 1085 Budapest, Hungary

**Keywords:** varicocele, male infertility, oxidative stress, DNA fragmentation, duplex ultrasonography, microsurgical varicocelectomy, non-obstructive azoospermia, hypogonadism, medically assisted reproduction

## Abstract

Varicocele is a common, potentially correctable condition associated with impaired male fertility. Despite being frequently encountered in clinical andrology, its pathophysiological mechanisms, diagnostic criteria, and therapeutic approaches remain areas of active investigation and debate. The authors conducted a comprehensive literature search, using the PubMed database, covering clinical studies, systematic reviews, meta-analyses, and current international guidelines from the past ten years. Emphasis was placed on studies investigating novel diagnostic modalities, therapeutic innovations, and prognostic markers. Emerging evidence supports the multifactorial pathophysiology of varicocele, involving oxidative stress, hypoxia, inflammatory pathways, and potential genetic predisposition. Biomarkers, including microRNAs, antisperm antibodies, and sperm DNA fragmentation, offer diagnostic and prognostic utility, though their routine clinical implementation requires further validation. Advances in imaging, such as shear wave elastography, may improve diagnostic accuracy. While microsurgical subinguinal varicocelectomy remains the gold standard, technological refinements and non-surgical alternatives are being explored. Indications for treatment have expanded to include selected cases of non-obstructive azoospermia, hypogonadism, and optimization for assisted reproduction, though high-level evidence is limited.

## 1. Introduction

Varicocele is one of the most prevalent and potentially correctable conditions, possibly associated with male infertility, affecting approximately 15% of healthy males and up to 35% of infertile men [1,2]. It is characterized by pathological dilation of the pampiniform plexus, leading to impaired venous drainage and varying degrees of venous reflux. These hemodynamic alterations contribute to increased scrotal temperature, hypoxia, oxidative stress, and the accumulation of toxic metabolites, with the risk of disruption of Sertoli and Leydig cell functions. These alterations can negatively impact spermatogenesis, reducing both sperm production and sperm quality [3], and can cause testicular pain. However, a substantial proportion of men with varicocele are asymptomatic and maintain normal fertility, and the causal relationship between varicocele and infertility remains a subject of ongoing debate [1,2].

The current European and American international guidelines [2,4,5] recommend diagnosing and grading varicocele based on the comprehensive medical history of the couple, physical examination, with ultrasound examination reserved for special circumstances. Grading consists of four grades, such as subclinical if not visible or palpable, but can be detected by ultrasound; grade I if it is palpable only during the Valsalva maneuver; grade II if it is palpable at rest; and grade III if it is visible [2].

Treatment is indicated in clinical varicocele (grades II-III) and testicular pain (excluding other possible causes). In terms of fertility issues, varicocele therapy is recommended for infertile relationships where semen parameters are abnormal, there are no gynecological factors, and the female partner has a preserved ovarian reserve. Idiopathic male infertility and elevated sperm DNA fragmentation are debated indication criteria. Surgical and radiological treatment options are available, but the microsurgical, subinguinal approach is the gold standard because of its effectiveness and low complication rate [2].

Despite recent advancements and evidence from clinical guidelines, numerous uncertainties persist regarding the pathophysiology, diagnostic evaluation, and optimal management of varicocele. In response, recent research efforts have increasingly focused on elucidating these aspects and generating an increasing body of literature resulting in higher-quality evidence.

The aim of this review is to summarize emerging trends and novel diagnostic and promising therapeutic modalities.

## 2. Materials and Methods

A comprehensive review of articles indexed in the PubMed database from the past ten years was conducted using the search term ‘varicocele.’ The selection encompassed all clinical studies, meta-analyses, and systematic reviews, along with additional publications and referenced works deemed relevant by the authors. Furthermore, current international clinical guidelines for the diagnosis and management of varicocele were also reviewed.

We summarize current international recommendations for the diagnosis and treatment of varicocele and highlight promising topics where available evidence is currently insufficient to support routine clinical implementation.

## 3. Potential Links Between Varicocele and Infertility

The proposed pathophysiological mechanisms underlying the detrimental effects of varicoceles on fertility include oxidative stress, elevated scrotal temperature, hypoxia, and accumulation of toxic metabolites. However, these factors do not appear to fully explain the entire phenomenon. With the advancement of diagnostic tools and techniques, additional theories have emerged regarding the common pathophysiological pathways linking varicocele to reduced male fertility.

### 3.1. Genetic and Molecular Aspects

The absence of a definitive causal relationship between varicocele and reduced male fertility or testicular pain suggests a multifactorial etiology and pathophysiology, potentially involving a genetic predisposition. The prevalence of varicocele is significantly higher among first-degree relatives of patients with grades II–III varicocele, reaching as high as 33.9%, supporting the hypothesis that a hereditary component contributes to the development of varicocele [6,7,8].

Genetic regulation of inflammation is well established, and as inflammatory pathways may be key to the pathophysiology of varicocele, their genetic basis is under investigation. Apelin, an anti-inflammatory peptide, produced by several tissues, including the hypothalamic–pituitary–gonadal axis, influences gonadotropin secretion and steroidogenesis. Akkan et al. found upregulated apelin and downregulated apelin receptor expression in the testicular tissue of varicocele-induced rats, indicating disrupted inflammatory signalling pathway [9].

Using whole-exome and transcriptome sequencing in rats and human (162 patients, 324 controls) cohorts, Yang et al. identified SPINT1, AAMP, and MKI67 as associated with varicocele [10]. Similarly, Zhang et al. used RNA and next generation DNA sequencing in varicose and non-varicose veins excised during varicocelectomy, and identified mutations in CFTR, NANOS1, SRCAP, GATA4, GCM2, TUBB1, ALDH7A1, ANTXR1, and MAP3K1 genes. Notably, CFTR mutations may impact fertility, and some genes are linked to alcohol metabolism, suggesting possible interactions between varicocele and alcohol consumption [11].

Karthikeyan et al. identified associations between varicocele and ARG2 and NOS1 mutations, implicating disrupted L-arginine metabolism and nitrosative stress in testicular dysfunction [12].

A meta-analysis by Mostafa et al. included 19 studies (1826 varicocele patients, 2070 controls, and 263 infertile men without varicocele) and found high heterogeneity and no conclusive genetic associations despite the analysis of GSTM1, GSTT1, NOS3, ACP1, NQO1, PRM1, PRM2, MTHFR, POLG, HSP90, and even mitochondrial genes [13].

Additionally, a systematic review by Naderi et al. suggested a potential link between varicocele and aberrant DNA/m64-RNA methylation, causing dysregulated oxidative stress responses [14].

Emerging evidence suggests a multifactorial genetic contribution to varicocele pathogenesis involving inflammatory, metabolic, and structural genes. However, further high-quality, large-scale studies are required to validate these associations and to delineate the underlying molecular mechanisms. A deeper understanding of the genetic architecture of varicocele may ultimately enable the development of targeted, non-surgical therapeutic interventions.

### 3.2. Seminal Fluid Analysis

Several studies have reported seminal fluid parameters in patients with varicocele. Longini et al. analyzed isoprostanoids in seminal plasma as they play a role in lipid peroxidation as potential oxidative stress biomarkers. Seminal F2-dihomo-isoprostanes, F2-isoprostanes, and F4-neuroprostanes were analyzed in three patient groups: fertile men, idiopathic infertile men, and infertile men with varicocele. Elevated isoprostanoid levels were significantly associated with increased sperm apoptosis, oxidative stress, and varicocele. These findings suggest that measurement of seminal isoprostanoids may help diagnose varicocele and identify candidates for surgery [15].

El Taieb et al. reported significantly elevated levels of seminal plasma leptin in 36 varicocele patients with asthenozoospermia compared to 30 fertile controls, with a marked reduction following surgical intervention. These findings suggest a potential pathophysiological association between varicocele and dysregulated carbohydrate metabolism. However, further research is warranted to substantiate this hypothesis [16].

A growing body of evidence highlights the role of microRNAs (miRNAs) as biomarkers in various pathologies, including testicular cancer, breast carcinoma, stroke, and pre-eclampsia. In the context of varicocele, Xu et al. [17] and Ma et al. [18] observed significantly increased expression of miR-210-3p in affected individuals. This miRNA was implicated in cell apoptosis and Sertoli cell dysfunction and showed a positive correlation with both varicocele grade and severity of infertility.

Although the use of miRNAs as diagnostic and prognostic biomarkers in andrology appears promising, their incorporation into routine clinical practice remains premature at this stage, pending further validation and standardization of detection methods.

### 3.3. Antisperm Antibodies

Antisperm antibodies (ASAs), often arising from blood–testis barrier disruption, have been widely studied; however, despite the theoretical and clinical relevance of this phenomenon, the number of high-quality systematic reviews and meta-analyses remains limited. A recent meta-analysis of six studies by Falcone et al. found significantly elevated ASA levels in the serum and seminal fluid of patients with varicocele, detected via both mixed antiglobulin reaction (MAR) and ELISA methodologies [19].

The WHO Laboratory Manual [20] recommends testing in cases of marked sperm agglutination, yet current guidelines from the European Association of Urology (EAU) and the American Urological Association (AUA) do not support its use for the evaluation of male infertility or varicocele [2,5].

Further research is needed to clarify the impact of ASAs on semen quality and fertility, to establish standardized indications and protocols for testing—particularly in cases of immune-related infertility and varicocele—and to support more informed decisions about varicocelectomy or medically assisted reproduction (MAR).

### 3.4. Sperm DNA Fragmentation (SDF)

Pathological alterations such as elevated scrotal temperature, hypoxia, oxidative stress, accumulation of toxic metabolites exert detrimental effects not only on Sertoli and Leydig cells but can also directly damage spermatozoa [21].

During oxidative stress, excessive accumulation of free radicals induces lipid peroxidation in the sperm plasma membrane as well as in the mitochondrial membrane, thereby reducing sperm motility and morphological quality. Due to their permeability, these free radicals can penetrate directly into the nucleus, causing direct damage to the DNA molecule and resulting in genetic and epigenetic alterations. This leads to increased DNA fragmentation, which is possibly associated with impaired sperm function [21].

Recent meta-analyses [21,22,23,24,25,26] have demonstrated a clear and strong association between the presence of clinically significant varicocele and increased DNA fragmentation. Furthermore, these studies have confirmed a significant reduction in DNA fragmentation following varicocele treatment.

In light of these findings, sperm DNA fragmentation (SDF) testing has been included in the European Association of Urology (EAU) guidelines as a recommended diagnostic tool in the evaluation of varicocele [2]. However, there is currently insufficient evidence to support its routine use in clinical practice.

## 4. Novel Trends in Diagnosis of Varicocele

Current clinical guidelines from the European Association of Urology (EAU), European Academy of Andrology (EAA), American Urological Association (AUA), and American Society of Reproductive Medicine (ASRM) primarily recommend the diagnosis of varicocele through physical examination, with ultrasound imaging reserved for specific clinical scenarios [2,4,5]. However, various ultrasound-based classification systems have been proposed (e.g., Hirsch, Sarteschi and Ligouri, European Academy of Andrology (EAA) classifications [27,28,29]), as shown in Table 1.

More recent studies, along with guidelines from the European Society of Urogenital Radiology, Scrotal and Penile Imaging Working Group (ESUR-SPIWG), advocate for the routine use of grey-scale and colour duplex ultrasound for varicocele diagnosis. These recommendations emphasize the need for both standardization and consistent implementation of ultrasound techniques, grading criteria, and uniform reporting protocol [30,31].

### 4.1. Grey-Scale Colour Duplex Ultrasound

Although grey-scale and colour duplex ultrasonography is not generally recommended to diagnose varicocele by European or American guidelines, except in cases of diagnostic uncertainty (obesity, hydrocele, cremaster spasm, etc.) or post-varicocelectomy, when semen analysis suggests incomplete surgery or recurrence [2], the European Society of Urogenital Radiology (ESUR) Scrotal and Penile Imaging Working Group (SPIWG) supports its routine use [30,31]. The discrepancy arises from differing clinical approaches. EAU and AUA/ASRM guidelines discourage the routine use of ultrasound as only palpable (grade II-III) varicoceles warrant treatment due to the lack of proven clinical benefit in lower grade cases. Consequently, physical examination is generally considered adequate for treatment planning unless the findings are uncertain [2,5]. In contrast ESUR-SPIWG advocates the routine use of ultrasound in clinical practice based on several factors including the potentially higher diagnostic accuracy of ultrasound [32,33], its ability to assess venous reflux—an important prognostic factor—and its utility in distinguishing relevant varicoceles from other conditions such as false varicocele (dilated veins without reflux) or varicocele-mimicking pathologies (cavernosus haemangiomas, lymphangiomas, arteriovenous malformations, etc.) [31,34].

A recent study by Jain et al. found that while physical examination identified only 15 men with varicoceles out of 70 infertile men, while ultrasound examination identified 30 men with varicocele in the same population, demonstrating its superior sensitivity [35].

Kayra et al. developed a machine-learning model for grading varicoceles based on data from 248 patients, emphasizing ultrasonographically measured vascular diameter and reflux duration with high accuracy (81%), sensitivity (0.69), and specificity (0.8). They recommended performing the examinations in the supine position and measuring vascular diameter at the testicular apex [36].

Recent ESUR-SPIWG guideline recommends ultrasonography over physical examination alone for accurate diagnosis and grading of varicocele [31]. In their publication in 2020, they also recommended standardized ultrasound documentation, including grey-scale and colour duplex data, vein diameter changes, and findings during standing and the Valsalva maneuver. The frequent coexistence of right-sided subclinical varicoceles in left-sided cases were also highlighted [30].

### 4.2. Shear-Wave Elastography

The improvement of sperm quality after varicocele surgery may be influenced by various less studied factors, including the number of veins ligated [37], the level of antioxidant protein expression preoperatively [38], and testicular characteristics such as volume and stiffness [39,40,41].

Increased testicular stiffness is associated with reduced sperm quality and concentration improvements after surgery. Shear wave elastography of the testes has been identified as a possibly useful predictive tool for sperm quality [42]. Patients with larger testicular volumes were more likely to experience greater improvements in sperm quality after varicocelectomy [37].

## 5. Novel Trends in the Management of Varicocele

**Figure 1:**
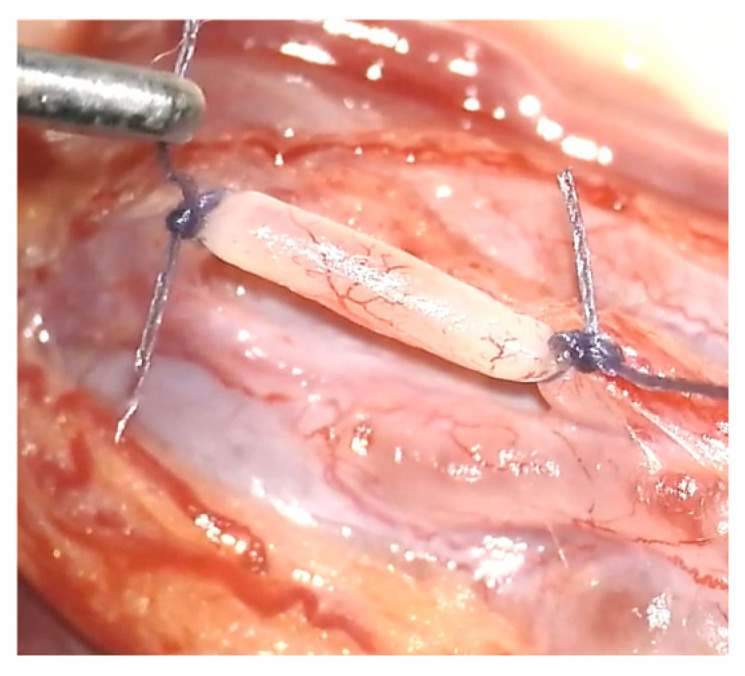
Ligated varicose vein during microsurgical subinguinal varicocelectomy.

Management of varicocele includes a variety of surgical techniques –such as open surgery, laparoscopy, and microsurgery—and can be performed using different approaches, including scrotal, inguinal, subinguinal, or retroperitoneal access. Additionally, interventional radiological options are available, including antegrade or retrograde sclerotherapy and retrograde embolization. Due to significant heterogeneity across studies in terms of patient populations, treatment modalities, and outcome measures, earlier meta-analyses yielded inconsistent findings regarding the efficacy of these interventions [43,44]. Radiological techniques have gained increasing popularity over the past decades, primarily due to advancements in technology and the associated shorter recovery times [45]. According to recent literature—including meta-analyses by Liu et al. (16 studies, 2138 patients) [46], Agarwal et al. (16 studies, 2420 patients) [47], Fallara et al. (12 studies, 1357 patients) [48], and a Cochrane Database systematic review by Persad et al. (5384 patients) [49]—fertility outcomes appear comparable between surgical and radiological interventions. While some analyses suggest a slight advantage for microsurgical techniques, this difference is not consistently observed across all meta-analyses. Improvements were primarily noted in sperm count and concentration, with less pronounced effects on motility and morphology. In terms of complication and recurrence rates, microsurgical approaches generally demonstrated superior outcomes compared to radiological techniques [2]. Among surgical approaches, the microsurgical subinguinal technique has emerged as the most effective, offering the lowest rates of complications and recurrence. Accordingly, it is endorsed as the gold standard by the European Association of Urology [2], supported by multiple studies [50,51] Figure 1.

### 5.1. Prognosis Assessment

At present, international guidelines do not contain specific information on predictive parameters that may influence surgical outcomes, particularly with regard to fertility. Despite the establishment of recent treatment indication criteria supported by a substantial body of scientific evidence, the prognostic accuracy for fertility improvement remains suboptimal. To enhance predictive precision, researchers continue to explore novel prognostic factors, expanded clinical indications, and emerging technological advancements.

#### 5.1.1. Preoperative Hormonal Parameters

Various studies have explored the role of hormonal and sperm analysis data in predicting fertility outcomes of varicocele treatment. Preoperative follicle-stimulating hormone (FSH) levels have been shown to correlate with postoperative sperm concentration [52,53], lower preoperative FSH levels were associated with better surgical outcomes, including improved sperm count and higher total motile sperm count (TMSC) [53,54]. Similarly, luteinizing hormone (LH) and total testosterone levels have been found to correlate with postoperative sperm concentration, further emphasizing the potential utility of hormonal profiles in predicting outcomes [52,53].

Inhibin B and insulin-like 3-peptide (INSL-3) have also been investigated for their predictive values. Lower preoperative levels of inhibin B correlate with more severe varicocele and were associated with lower sperm concentration and morphology postoperatively [55]. These findings suggest that hormonal parameters may play a role in predicting the outcomes of varicocelectomy, particularly in terms of fertility.

#### 5.1.2. Results of Preoperative Ultrasound and Semen Analysis

The grade of left varicocele is a significant predictor of postoperative sperm concentration, motility, and total motile sperm count (TMSC) [56]. High-grade varicoceles show greater improvement in sperm concentration after varicocelectomy, especially when venous diameters exceed 5 mm [57]. Additionally, increased reflux correlates with worse sperm concentration and motility, highlighting its detrimental effect on semen quality [58].

A meta-analysis by Ou et al. found that bilateral varicocelectomy, including cases of concomitant clinical left and subclinical right varicoceles, yields better outcomes—especially in motility—than unilateral repair [59]. However, other meta-analyses suggest that treating subclinical varicoceles alone results in minimal or no improvement [60,61], Ou et al. supported a combined approach for better results [59].

Baseline sperm concentration also influences surgical outcomes. Patients with severe oligozoospermia often experience little or no improvement postoperatively, whereas those with normal or moderately reduced concentration and motility may benefit more [62]. Moreover, lower sperm concentration is linked to higher rates of genetic abnormalities, which may limit the benefits of varicocelectomy [63].

Preoperative TMSC and progressive motility are also useful predictors of postoperative improvement [54,56,64]. Shabana et al. used ROC curve analysis to define responders to surgery as those with sperm concentrations >8 million/mL and progressive motility >18% [65].

In fertility outcomes, higher spontaneous pregnancy rates have been associated with TMSC and pre-surgery progressive motility >30% [66], although it does not apply for patients with severe oligozoospermia [67]. Bilateral varicocelectomy improves chances of spontaneous pregnancy and live birth rate more than unilateral repair [59,68], especially when reflux resolves postoperatively [69].

Despite these findings, outcome prediction remains inconsistent, and no single parameter offers universal reliability for all patients with varicocele-associated infertility.

#### 5.1.3. Scoring Systems, Nomograms, and Inflammatory Markers in Outcome Prediction

Identifying ideal candidates for varicocelectomy and predicting therapeutic outcomes remain significant clinical challenges. As summarized by Crafa [70] and discussed in previous chapters, a comprehensive assessment of seminal parameters, endocrine status, and evaluation of seminal fluid parameters may support not only diagnosis but also outcome prediction.

In this context, Ory et al. developed a machine learning model to predict fertility outcomes after varicocele repair. Using artificial intelligence (AI) that incorporates clinical, seminal, and hormonal data from 240 patients with clinically significant varicoceles and abnormal semen parameters, the model achieved an 86.7% accuracy in predicting clinically meaningful fertility improvements, outperforming traditional methods [71].

Similarly, Maimaitiming et al. constructed a predictive nomogram using multivariate data from 268 patients, including age, BMI, varicocele grade, testicular atrophy, elastography, testosterone, and semen parameters–. Treatment success was defined as a 25% improvement in semen parameters. The nomogram showed strong predictive power, with AUC values of 0.915 for total sperm count, 0.986 for concentration, and 0.924 for vitality [72].

Systemic inflammatory markers have also shown prognostic value. Duran et al. and Ates et al. found that elevated monocyte-to-lymphocyte (MLR) and neutrophil-to-lymphocyte ratios (NLR) are associated with poorer postoperative semen outcomes [73,74]. Erdogan et al. further identified a negative correlation between varicocelectomy success and inflammatory indices such as SIRI (systemic inflammatory response index) and SII (systemic immune-inflammation index) in a cohort of 207 patients [75].

In addition, Liu et al. proposed a nomogram to predict spontaneous conception after microsurgical varicocelectomy, using predictors such as age of female partner, venous diameter, and changes in total progressively motile sperm count (TPMSC), based on data from 282 patients [76].

These emerging tools—including AI models, nomograms, and inflammatory markers—offer promising strategies for personalized management of varicocele-associated infertility. However, large-scale prospective trials are needed before routine clinical adoption.

### 5.2. New Indications of Varicocele Treatment

According to the recent European Association of Urology Guidelines on Sexual and Reproductive Health, varicocele treatment is recommended in cases of clinically palpable varicocele (grade II or III), with abnormal conventional semen parameters, otherwise unexplained infertility, and preserved ovarian reserve in the female partner with strong recommendation. Additionally, varicocele repair may be considered in men with elevated sperm DNA fragmentation and a history of failed assisted reproduction procedures, though evidence remains limited [2].

Treatment of subclinical varicocele is not recommended in the absence of a contralateral clinical varicocele as current evidence does not demonstrate relevant clinical benefit [2]. In adolescent males, intervention is only recommended in cases of progressive testicular atrophy, defined by a testicular volume discrepancy exceeding 2 mL or 20%, consistent with EAU recommendations [2].

Despite the clarity of current clinical guidance, recent studies have proposed additional indications for varicocele repair, including the management of non-obstructive azoospermia, in the enhancement of MAR outcomes, and, more recently, in the treatment of hypogonadism. Although these indications are acknowledged by the EAU Guidelines, they are not yet universally adopted in other guideline frameworks and lack robust, high-quality evidence to support routine clinical implementation.

#### 5.2.1. Non-Obstructive Azoospermia (NOA)

The weak evidence supporting the clinical benefit of varicocele repair in non-obstructive azoospermia (NOA) primarily comes from case series and small cohort studies [77]. A comprehensive meta-analysis by Estevez et al. of 18 studies, encompassing 468 patients, found a significantly higher sperm retrieval rate (SRR) in treated patients compared to untreated controls (OR: 2.65; *p* < 0.001), although improvements in clinical pregnancy and live birth rates were not statistically significant (clinical pregnancy: *p* = 0.08; live birth *p* = 0.05). In 15 studies evaluating post-treatment ejaculates, spermatozoa were present in 43.9% of patients [78].

Abdel-Meguid et al. reported a positive SRR of 32.3% in a cohort of 31 NOA patients after varicocele surgery, although intermittent sperm presence was observed in 13% of these cases in the ejaculate [79].

The effectiveness of varicocele repair in NOA patients depends on testicular histopathology, with better results in hypospermatogenesis or late maturation arrest, and poor outcomes in Sertoli cell-only syndrome [79,80,81].

A more recent meta-analysis by Ramon et al. including 9 studies and more than 1170 patients, showed a SRR of 15–62.5% in treated patients versus 0–19% in controls [82]. Similar findings were reported by Jensen et al. in a systematic review; however, they also highlighted a notable relapse rate of azoospermia post-varicocelectomy (20.8%) [81].

Overall, varicocele repair may be beneficial for some NOA patients as it may allow natural sperm recovery and potentially avoids surgical retrieval. However, benefits may be transient, the quality of evidence is limited, and routine use is not currently recommended without careful patient selection and counselling.

#### 5.2.2. Medically Assisted Reproduction (MAR)

Although varicocelectomy and medically assisted reproduction (MAR) are both commonly used to treat male infertility, relatively few randomized controlled trials (RCTs) have assessed the impact of varicocele repair on MAR outcomes. While improvements in semen parameters after varicocelectomy are well documented, its effect on MAR success remains unclear, however recent guidelines from the European Association of Urology (EAU) and American Urological Association (AUA) recommend varicocele treatment not only to potentially avoid the need for MAR, but also to improve its outcomes [2,5].

A recent meta-analysis by Palani et al. analyzed nine observational studies comparing treated and untreated varicocele patients undergoing various MAR procedures. For IUI, no significant difference in clinical pregnancy rates was found, but one study reported a significantly higher live birth rate in the treated group (*p* = 0.007). In ICSI, varicocele repair was associated with significantly higher fertilization (*p* < 0.01), clinical pregnancy (*p* = 0.01), and live birth rates (*p* < 0.01) [83]. These findings align with prior meta-analyses by Kirby et al. and Esteves et al. that reported improved outcomes in treated men, although implantation and miscarriage rates remained inconclusive [84,85]. A major limitation of these studies was the heterogeneity in inclusion and exclusion criteria, study design, and outcome measures.

Varicocele repair may offer clinical benefits before or instead of MAR, but improvement typically takes 6 months postoperatively. Therefore, individualized treatment planning, including a thorough discussion of expectations and timing, is essential. Jayadevan et al. found that men who received targeted counselling showed less decisional conflict and often switched from IVF to varicocelectomy [86].

When properly indicated, varicocele treatment can enhance MAR success or reduce the need for it. However, treatment decisions should be made after thorough consideration of factors of the female partner (e.g., ovarian reserve), and a well-informed consent process. Guidelines also emphasize the need for further large-scale, high-quality RCTs to better elucidate the clinical benefits of varicocele repair in the context of MAR.

#### 5.2.3. Hypogonadism

Although Leydig cells are relatively more resistant, they remain vulnerable to the same pathological factors that affect Sertoli cells and spermatozoa. These can impair enzymatic activity and trigger apoptosis in Leydig cells [87,88], making varicocele a potential risk factor for hypogonadism.

This association has been recognized in the European Association of Urology Guidelines on Sexual and Reproductive Health since 2020. However, the available meta-analytic evidence remains limited [2].

A meta-analysis by Chen et al. of 8 studies (712 patients) found a significant increase in serum testosterone level after varicocelectomy—34.3 ng/dL overall, (*p* < 0.001), and 123 ng/dL in hypogonadal men (*p* < 0.001), with no notable change in eugonadal individuals [89].

Similarly, Gonzalez-Daza et al. reviewed ten studies in their meta-analysis and reported a higher prevalence of hypogonadism in varicocele patients (OR: 3.27) [87]. Cannarella et al. conducted the most extensive meta-analysis to date (48 studies, 4/48 randomized trials, more than 4100 patients), and reported a significant increase in testosterone levels postoperatively (mean difference: 82.45 ng/dL, *p* < 0.00001), and compared to untreated controls (91.64 ng/dL, *p* < 0.00001), although levels did not significantly differ from those in healthy men (*p* = 0.35) [90]. These results are supported by Cayan et al. and Tian et al., both demonstrating that testosterone levels increase after varicocele treatment [88,91].

Despite consistent findings, the underlying studies vary widely in quality, design, and follow-up, with many being observational. As such, evidence should be interpreted with caution. Recommendations for varicocele treatment as a treatment for hypogonadism remain debated due to a lack of standardized, high-quality data and unclear criteria for patient selection.

The EAU Guidelines suggest that varicocelectomy may be offered to hypogonadal men with clinical varicocele (grade II-III) following thorough counselling about alternatives, including testosterone therapy and cost-effectiveness [2]. In contrast, the AUA/ASRM Guidelines currently provide no specific recommendations [5].

### 5.3. Novel Techniques and Methods in Varicocele Treatment

#### 5.3.1. Stem Cell Therapy

Stem cell therapy represents a promising advance in regenerative medicine and is already in clinical use for erectile dysfunction, with experimental applications for azoospermia [92].

Serefoglu et al. evaluated the effects of varicocelectomy versus intratesticular injection of adipose-derived mesenchymal stem cell-conditioned medium (ADMSC-CM) in a rat model. No significant differences were observed in semen parameters, markers of oxidative stress (malondialdehyde, superoxide dismutase), or blood–testis barrier integrity (Claudin-11), indicating comparable efficacy between the two treatments [93].

Peserico et al. investigated the intratesticular injection of human amniotic fluid mesenchymal stromal cells (hAFMSCs) and amniotic epithelial cells (hAECs) in a rat model of varicocele. hAECs reduced inflammation, while hAFMSCs supported Sertoli cell function. Both cell types modulated the testicular endocannabinoid system toward a pro-regenerative state, suggesting potential for fertility restoration. These findings highlight stem cell therapy as a promising, cell-specific, non-surgical approach to testicular regeneration [94], but perhaps it is over-emphasized in relation to current applicability.

#### 5.3.2. Surgical Techniques

Several surgical innovations have been introduced to improve the safety and efficacy of varicocelectomy, particularly to reduce recurrence rates and complications such as hydrocele formation. One such approach is the use of indocyanine green (ICG) fluorescence lymphography to spare lymphatic vessels during laparoscopic varicocelectomy. Fuente et al. studied 30 adolescents laparoscopic varicocelectomy patients, dividing them into an ICG group (*n* = 13) and a non-ICG group (*n* = 17). In the non-ICG group, nearly one-third of the patients developed postoperative hydroceles, and one patient required reoperation, while no such complications occurred in the ICG group. Importantly, no recurrences were observed in either group during the one-year follow-up [95]. Esposito et al. expanded on this technique in a larger study of 72 adolescents with high-grade (grade II or III) varicoceles, demonstrating that ICG-guided laparoscopic lymphatic-sparing varicocelectomy was both safe and effective. No allergic reactions were reported, hydrocele formation was completely avoided, and only a single hematoma occurred. Three cases of intratesticular calcifications were observed at the site of ICG injection, two of which resolved over time. However, varicose veins persisted in 4 patients, and 15 of 42 boys operated on for ipsilateral testicular growth retardation failed to exhibit any catch-up growth postoperatively [96]. This technique seems unnecessary with microsurgical approach, as risk of postoperative hydrocele is minimal (0.44%) [2].

Other intraoperative technologies have also enhanced surgical outcomes. Hashim et al. employed a video telescopic operating microscope (VITOM) in 23 adult patients with high-grade varicoceles and symptoms of testicular pain or swelling. Using a subinguinal approach and 16-fold magnification, they were able to clearly identify arterial pulsation and spare arteries. No postoperative complications were reported. Only one patient experienced persistent pain, which was milder than the preoperative level [97]. Duarsa et al. reported 8.6% scrotal edema and 2.9% hydrocele formation using the same technique on 35 varicocele patients [98].

Kaya et al. utilized intraoperative microvascular duplex ultrasonography during subinguinal microsurgical varicocelectomy in 19 adolescents—10 with testicular hypotrophy and 9 with pain. The study found that microscopic visualization alone was often insufficient to detect testicular arterial pulsation, whereas duplex made this significantly easier. No complications were observed, and six of the ten hypotrophic testes showed catch-up growth within one year [99]. Lv et al. reported significantly better vein identification and postoperative pain resolution (*p* = 0.033) after microsurgical varicocelectomy using intraoperative microvascular duplex ultrasonography [100].

In contrast, testicular delivery during varicocelectomy—a technique intended to reduce recurrence by facilitating ligation of gubernacular veins—has not demonstrated consistent benefits. Ramasamy et al. retrospectively analyzed 165 patients (55 with and 110 without testicular delivery) and found no significant differences in fertility, hypogonadism, or recurrence [101]. Wald et al. assessed 313 patients and also found no difference in sperm improvement. However, there was a non-significant trend toward higher postoperative testosterone levels in the testicular delivery group (*p* = 0.06) [102]. Jin et al. evaluated 181 patients and actually found better sperm concentration and testosterone levels in those who had not undergone testicular delivery, particularly in grade III varicocele cases [103]. Hou et al. reported similar findings [104]. In paediatric cases, Choi et al. observed a higher recurrence rate in 58 patients who underwent testicular delivery [105]. A meta-analysis of 8 studies and 1139 patients by Song et al. confirmed that testicular delivery does not improve fertility outcomes and is associated with a higher risk of complications, such as orchidoepididymitis and scrotal edema, as well as increased operative time [106]. As a result, current clinical guidelines do not recommend testicular delivery.

Robotic-assisted varicocelectomy has also gained attention due to its enhanced precision, tremor reduction, and 3D visualization, though its adoption is limited by high cost, the need for general anaesthesia, and lack of definitive evidence for superiority. A narrative review by Napolitano et al. emphasized the absence of randomized controlled trials supporting the robotic approach over traditional methods [107]. Early work by Shu et al. comparing eight robotic-assisted and eight conventional microsurgical procedures found no significant differences in operative time or complication rates [108]. However, in a larger retrospective study of 258 robotic-assisted subinguinal procedures, McCullough et al. reported a 37.3% improvement in sperm concentration (*p* < 0.03), a mean testosterone increase of 145 ng/dL (*p* < 0.001), and low complication rates (hydrocele: 0.8%, hematoma: 2.7%) [109]. Similarly, Parekattil et al. found that 77% of patients had improved sperm counts and 96% reported pain relief postoperatively [110]. While these findings are promising, the robotic technique remains an expensive and technically demanding option without definitive proof of superior outcomes over conventional microsurgical varicocelectomy.

#### 5.3.3. Antioxidant Treatment

Oxidative stress is a central pathophysiological factor in male infertility associated with varicocele, underpinning the rationale for antioxidant supplementation aimed at mitigating oxidative damage and improving fertility outcomes. Despite widespread clinical use, evidence of its efficacy remains inconclusive.

Pyrgidis et al. analyzed 14 studies (*n* = 980) and found no significant improvement in pregnancy rates with antioxidants; results on semen parameters and sperm DNA fragmentation were inconsistent, hindered by methodological heterogeneity [111]. Ioannidou et al. focused on non-operated varicocele patients (6 studies, *n* = 213), reporting significant improvements in sperm concentration, progressive motility, and seminal volume, but not morphology [112]. Agarwal et al. reviewed 97 studies including 11 on varicocele; only 2 were high-quality RCTs, concluding antioxidants yielded non-significant improvements in semen quality and sperm function [113].

Wang et al. examined post-varicocelectomy antioxidant therapy in six RCTs (*n* = 576) and found significant improvement in sperm concentration, motility, morphology, and reduced levels of FSH at 3 and 6 months, yet no increase in pregnancy rate [114]. Similarly, Chen et al. (2018) reported improved sperm parameters and DNA integrity after 3 months of antioxidants in 10 RCTs (*n* = 901), without corresponding fertility benefits [115].

In conclusion, meta-analyses consistently indicate that antioxidant therapy does not significantly improve pregnancy rates in varicocele-associated infertility. Although some semen quality parameters may improve, findings are inconsistent and limited by study heterogeneity and quality. High-quality, large-scale RCTs are warranted to define the clinical value of antioxidant supplementation in this context.

## 6. Conclusions

Varicocele is a common condition with significant implications for male fertility, making its diagnosis and management a central concern in andrology and urology. This review highlights recent advancements in pathophysiology, diagnostic, and therapeutic strategies. Grey-scale and duplex ultrasound remain the primary imaging modalities for diagnosis, while emerging techniques such as shear wave elastography, seminal fluid analysis, and detection of antisperm antibodies may offer supplementary diagnostic value. Optimal surgical outcomes are contingent on appropriate patient selection, with prognostic evaluation enhanced by preoperative ultrasound, semen analysis, hormonal profiling, integration of artificial intelligence (AI) models and clinical nomograms. Expanding indications for varicocele repair—including non-obstructive azoospermia (NOA), hypogonadism, and optimization for assisted reproductive technologies (ART/MAR)—are promising but require further validation through large-scale randomized controlled trials. Antioxidant therapy may serve as a beneficial adjunct in selected cases. Technological innovations continue to replace (stem cell therapy, antioxidant therapy) or refine surgical techniques, enhancing precision and reducing complication rates; however, the clinical utility of robotic-assisted varicocelectomy remains limited due to high costs and unproven superiority, which keeps the microsurgical approach as a gold standard procedure. Notably, testicular delivery during surgery appears to increase the risk of complications without offering clear clinical benefits.

## Figures and Tables

**Table 1:** Ultrasonographic classifications of varicocele.

Classification	Basis of Grading	Grade I	Grade II	Grade III	Grade IV	Grade V
**Hirsch (1980)** [28]	Reflux pattern	No spontaneous reflux, elicitable with Valsalva maneuver.	Intermittent spontaneous reflux.	Continuous spontaneous reflux.	-	-
**Sarteschi (1993)** [27]	Vein diameter, reflux pattern	No dilated veins, only inguinal reflux during Valsalva maneuver.	Supra-testicular varicosities, reflux during Valsalva maneuver.	Peritesticular varicosities only in standing position, reflux during Valsalva maneuver.	Dilated veins in supine position, increasing in standing position and Valsalva, reflux at rest, increasing with during Valsalva maneuver, possible testicular hypotrophy.	Dilated veins in standing and supine position, reflux at rest, not increasing during Valsalva maneuver.
**EAA (Lotti, 2022)** [29]	Vein diameter, reflux pattern	Dilated veins (>3 mm) at rest at the funicular region with retrograde venous flow absent/intermittent at rest and enhanced during Valsalva maneuver.	Dilated veins (>3 mm) at rest at the upper pole of the testis with retrograde venous flow absent/intermittent at rest and enhanced during Valsalva maneuver.	Dilated veins (>3 mm) at rest at the lower pole of the testis with retrograde venous flow absent/intermittent at rest and enhanced during Valsalva maneuver.	Dilated veins (>3 mm) at rest (irrespective of location but usually extending to the peritesticular region) with retrograde venous flow continuous at rest and enhanced during Valsalva maneuver. Possible testicular hypotrophy.	Dilated veins (>3 mm) at rest (irrespective of location, but usually extending to the peritesticular region) with retrograde venous flow continuous at rest and not increasing during Valsalva maneuver.

## Abbreviations

ASA	Antisperm Antibodies
AI	Artificial Intelligence
AUA	American Urological Association
AUC	Area Under the Curve
ARTs	Assisted Reproductive Technologies
ASRM	American Society for Reproductive Medicine
CFTR	Cystic Fibrosis Transmembrane Conductance Regulator
DNA	Deoxyribonucleic Acid
EAU	European Association of Urology
EAA	European Academy of Andrology
ESUR-SPIWG	European Society of Urogenital Radiology-Scrotal and Penile Imaging Working Group
FSH	Follicle-Stimulating Hormone
hAFMSCs	Human Amniotic Fluid Mesenchymal Stromal Cells
hAECs	Human Amniotic Epithelial Cells
ICG	Indocyanine Green
INSL-3	Insulin-like 3
IUI	Intrauterine Insemination
ICSI	Intracytoplasmic Sperm Injection
LH	Luteinizing Hormone
MAR	Medically Assisted Reproduction
MLR	Monocyte-to-Lymphocyte Ratio
miRNA	MicroRNA
NOA	Non-Obstructive Azoospermia
NLR	Neutrophil-to-Lymphocyte Ratio
ROC	Receiver Operating Characteristic
SDF	Sperm DNA Fragmentation
SII	Systemic Immune-Inflammation Index
SIRI	Systemic Inflammatory Response Index
SRR	Sperm Retrieval Rate
TMSC	Total Motile Sperm Count
TPMSC	Total Progressively Motile Sperm Count
VITOM	Video Telescopic Operating Microscope
WHO	World Health Organization
